# Cost-Effectiveness Analysis of Direct Oral Anticoagulants Vs. Vitamin K Antagonists in the Elderly With Atrial Fibrillation: Insights From the Evidence in a Real-World Setting

**DOI:** 10.3389/fcvm.2021.675200

**Published:** 2021-06-29

**Authors:** Yue Wu, Chi Zhang, Zhi-Chun Gu

**Affiliations:** ^1^Department of Pharmacy, Renmin Hospital, Wuhan University, Wuhan, China; ^2^School of Pharmaceutical Sciences, Wuhan University, Wuhan, China; ^3^Department of Pharmacy, Renji Hospital, School of Medicine, Shanghai Jiao Tong University, Shanghai, China; ^4^School of Medicine, Tongji University, Shanghai, China; ^5^Shanghai Anticoagulation Pharmacist Alliance, Shanghai Pharmaceutical Association, Shanghai, China; ^6^Chinese Society of Cardiothoracic and Vascular Anesthesiology, Beijing, China

**Keywords:** atrial fibrillation, elderly, direct oral anticoagulants, cost-effectiveness, real-world study, edoxaban, rivaroxaban, dabigatran

## Abstract

**Background:** In the clinical setting, the economic benefits of direct oral anticoagulants (DOACs) in elderly patients with atrial fibrillation (AF) remain unclear. This study aimed to estimate and compare the cost-effectiveness of DOACs (dabigatran, rivaroxaban, apixaban, and edoxaban) and vitamin K antagonists (VKAs; warfarin) in preventing stroke among AF patients aged >75 years in real-world practice.

**Methods:** A Markov model with a 10-year span was constructed to estimate the long-term clinical and economic outcomes among AF patients aged >75 years treated with DOACs and warfarin. The study was populated with a hypothetical cohort of 10,000 AF patients aged >75 years. Probabilities of clinical outcomes were obtained from the pooled observational studies (OSs), comparing DOACs (dabigatran, rivaroxaban, apixaban, and edoxaban) with VKAs. Other model inputs, including the utilities and the costs, were all estimated from public sources and the published literature. The costs, quality-adjusted life-years (QAYLs), and incremental cost-effectiveness ratios (ICER) were estimated for each treatment strategy. Subgroup analyses of individual DOACs and the scenario analysis were performed. Uncertainty was evaluated by deterministic sensitivity analysis and probabilistic sensitivity analysis (PSA).

**Results:** Compared to warfarin, DOACs were associated with a gain of 0.36 QALY at an additional cost of $15,234.65, resulting in an ICER of $42,318.47 per QALY. Sensitivity analysis revealed that the ICER was sensitive to the cost of DOACs. Direct oral anticoagulants also shifted from dominating to dominated status When their annual costs of DOACs were over $3,802.84 or the risk ratio of death compared to warfarin was over 1.077%/year. Probabilistic sensitivity analysis (PSA) suggested that DOACs had a 53.83 and 90.7% probability of being cost-effective when the willingness-to-pay threshold was set at $50,000 and $100,000, respectively. Among all the four individual DOACs, edoxaban treatment was revealed as the preferred treatment strategy for the AF patients aged over 75 years by yielding the most significant health gain with the relatively low total cost.

**Conclusions:** Despite the high risk for major bleeding in elderly patients with AF, DOACs are more cost-effective treatment options than warfarin in real-world practice. Edoxaban was the preferred treatment strategy among four kinds of DOACs for AF patients aged over 75 years. Furthermore, beyond their safety profiles, the treatment benefits of DOACs assumed greater relevance and importance in older adults.

## Introduction

Atrial fibrillation (AF) is the most common type of cardiac arrhythmia in adults, affecting approximately 33 million individuals worldwide (1). Epidemiological studies have shown that advancing age is a major risk factor for AF, with its prevalence almost doubling with every 10-year increase in age (from 0.1% among patients aged <35 years to 14% among those aged >75 years) (2–4). Age is also a non-modifiable risk factor for ischemic stroke in patients with AF. More than 50% of ischemic stroke cases diagnosed in patients with AF occur in those aged >80 years (5). Given the high mortality and disability rates associated with AF-induced ischemic stroke, it is considered major public health, social, and economic burden in the elderly population (6).

Traditionally, stroke in AF has been prevented by using vitamin K antagonists (VKAs), such as warfarin. Owing to their efficacy profiles, which have been recently reported by several large, randomized controlled trials (RCTs), direct oral anticoagulants (DOACs), such as dabigatran, rivaroxaban, apixaban, and edoxaban, are viable alternatives for VKAs and have been established as cornerstones in AF management for the prevention of stroke (7, 8). Based on the results of a meta-analysis of RCTs, DOACs have been reported to be more cost-effective than VKAs (9–11). However, since no RCT was designed to focus on the elderly population specifically, there is little clarity regarding the health and economic benefits of DOACs in the said population (12). Although subgroup analyses of RCTs have usually been conducted for the elderly population, there remain many uncertainties about the relevance of the results of these RCTs in the real-world setting, given that the elderly population is known in real practice to be at high risk for bleeding (13). Furthermore, given the poor prognosis and heavy burden of major bleeding events in elderly patients with AF, it remains unknown whether the benefits of DOACs will be offset by the high incidence rates of intracranial bleeding in real clinical settings. Therefore, using data from a comprehensive meta-analysis of high-quality OSs and RCTs, we performed a cost-effectiveness evaluation comparing DOACs and VKAs to assess the expected costs and benefits of using the former in the elderly population with AF in real-world settings.

## Methods

### Model

**Figure 1:**
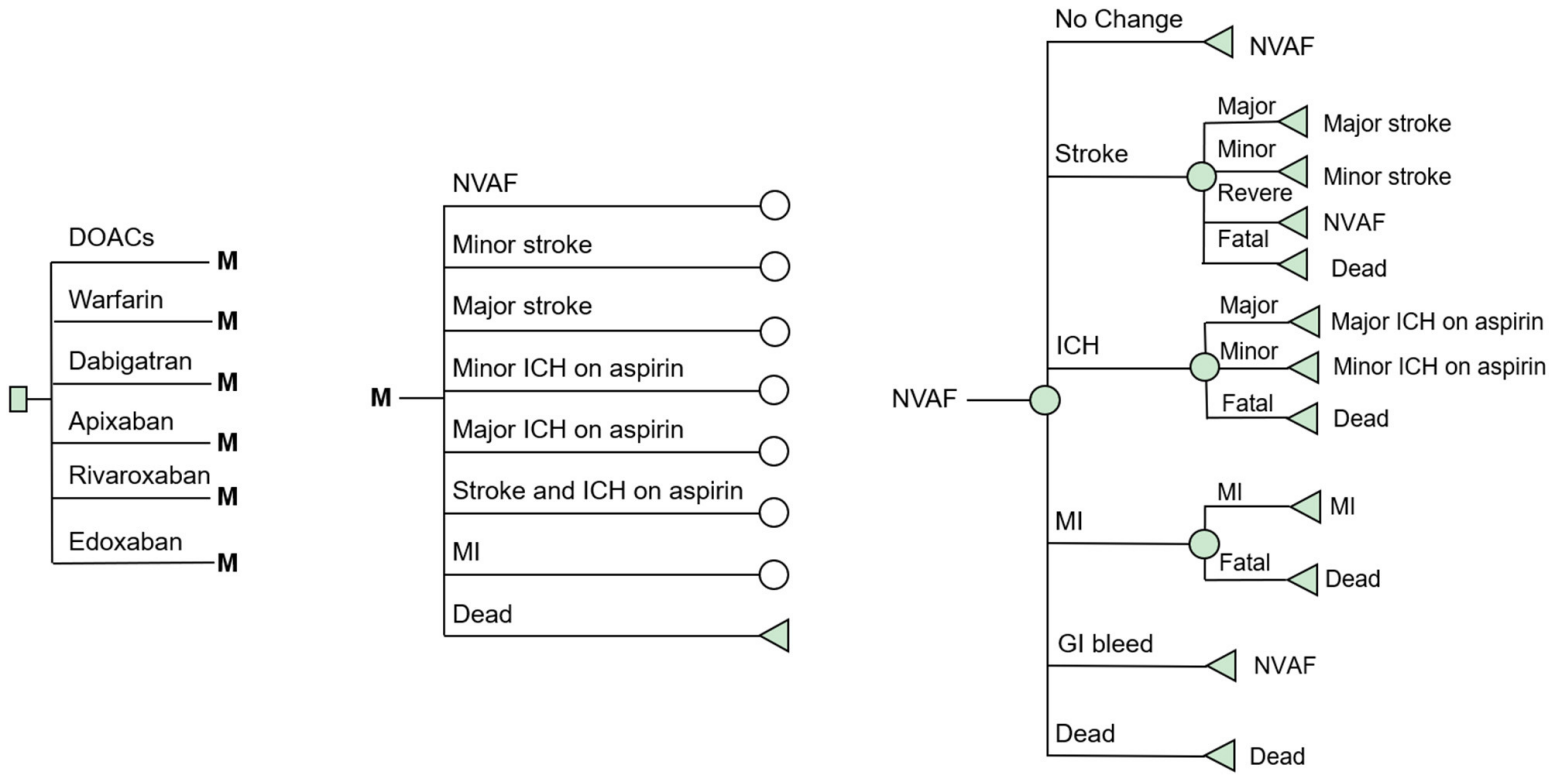
Schematic representation of the Markov model. AF, atrial fibrillation; Stroke, ischemic or hemorrhagic stroke; SE, systemic embolism; GI Bleeding, gastrointestinal bleeding; ICH, intracranial hemorrhage; MI, myocardial infarction.

A decision-analysis Markov model with a 1-year cycle and a 10-year horizon was constructed to compare the cost-effectiveness of DOACs (including dabigatran, apixaban, rivaroxaban, and edoxaban) and VKAs in elderly AF patients in real-world settings. A simplified schematic represents the model structure in Figure 1. Eight mutually exclusive health states were included in the Markov model: AF without complications, major ischemic stroke, minor stroke, major intracranial hemorrhage (ICH) on aspirin, minor ICH on aspirin, stroke and ICH on aspirin, myocardial infarction (MI), and death. The following assumptions were made to reflect the approximate progression of AF in older age: The starting age of the patient cohort was set at 75 years, and all were assumed to begin in the state of AF without complications. In each cycle, patients may either remain in their current health state or transition to the next due to a clinical event. Four types of stroke (reversible, major, minor, and fatal) and three types of ICH (major, minor, and fatal) were included in the model. To reflect the treatment pattern of AF and to approximate the AF disease progression in real-world practice, we made the following assumptions in the model. Due to the high case fatality rate in the hospitalized stroke, which reflects the immediate severity of the condition, transition to death was assumed after two major neurological events (stroke or ICH) (14, 15). Patients with two minor neurological were supposed to proceed to the major event state (15). After any ICH, patients were assumed to discontinue the therapy of anticoagulants and switch to aspirin in case of recurrent ICH. This estimate of drug switch is widely used in previously published cost-effectiveness analyses of anticoagulants (15–17). Considering that the risk of MI is usually greatly reduced with anticoagulant therapy, no MI event was considered after any neurological events. TreeAge Pro Suite TM software 2019 (Williamstown, MA. USA) was used for the model construction and analysis.

### Date and Sources

#### Treatment Effectiveness

A targeted literature review was performed to identify the appropriate parameter inputs. The probabilities of the clinical outcomes associated with VKAs were obtained from the included OSs or RCTs, respectively (Table 1 and Supplementary Table 1). The comparative probabilities of clinical outcomes relative to DOACs were calculated based on the corresponding rates of VKAs and the pooled hazard ratios reported in our previous work (21) (Table 1 and Supplementary Table 1). The relative risks for clinical outcomes for aspirin compared to warfarin were obtained from a network meta-analysis reported previously (34). The age-dependent baseline death rates for stroke, ICH, and MI were derived from the Global Burden of Disease Study 2017 (35), which were assumed to be similar in DOACs and VKAs (Table 2). All clinical event rates were ultimately converted into probabilities per cycle and then input into the model.

**Table 1:** Parameters inputs.

**Parameters**	**Base-case value**	**Low**	**Up**	**Distribution**	**Sources**	**Notes**
**PROBABILITY**						
Stroke (warfarin)	0.0081	0.00648	0.00972	Beta	Zoppellaro et al. (18)	Assumption range of ±20%
ICH (warfarin)	0.0129	0.0103	0.0387	Beta	Zoppellaro et al. (18)	Assumption range of −20–300%
MI (warfarin)	0.0169	0.0135	0.0203	Beta	Graham et al. (19)	Assumption range of ±20%
GI bleeding (warfarin)	0.009	0.0072	0.027	Beta	Zoppellaro et al. (18)	Assumption range of −20–300%
Mortality (warfarin)	0.0378	0.0303	0.0454	Beta	Graham et al. (19)	Assumption range of ±20%
Proportion of major ICH	0.141	0.009	0.241	Beta	Wang et al. (15)	95%CI
Proportion of minor ICH	0.495	0.396	0.594	Beta	Wang et al. (15)	95%CI
Proportion of fatal MI	0.166	0.158	0.174	Beta	Krumholz et al. (20)	95%CI
Proportion of major stroke	0.402	0.402	0.417	Beta	Wang et al. (15)	95%CI
Proportion of minor stroke	0.425	0.348	0.425	Beta	Wang et al. (15)	95%CI
Proportion of reversible stroke	0.091	0.091	0.133	Beta	Wang et al. (15)	95%CI
**THE RELATIVE RISK OF DOACS RELATIVE TO WARFARIN (OSS/RCT)**						
Stroke (OSs/RCT)	0.87/0.82	0.81/0.67	0.94/0.96	Beta	Shen et al. (21)	Meta-analysis, 95% CI
ICH (OSs/RCT)	0.47/0.47	0.37/0.31	0.57/0.63	Beta	Shen et al. (21)	Meta-analysis, 95% CI
MI (OSs)	0.89	0.79	0.99	Beta	Shen et al. (21)	Meta-analysis, 95% CI
GI bleeding (OSs/RCT)	1.21/1.34	0.98/0.91	1.43/1.77	Log-normal	Shen et al. (21)	Meta-analysis, 95% CI
Mortality (OSs/RCT)	1.01/0.94	0.92/0.87	1.11/1.00	Log-normal	Shen et al. (21)	Meta-analysis, 95% CI
**UTILITY**						
AF	0.81	0.7	0.9	Beta	Sullivan et al. (22)	95% CI, ICD-9 427
**UTILITY DECREMENT**						
Minor stoke or ICH	0.2916	0.2800	0.3000	Beta	Tengs et al. (23)	95% CI
Major stroke or ICH	0.4455	0.4300	0.4600	Beta	Tengs et al. (23)	95% CI
Reversible stroke	0.2916	0.2800	0.3000	Beta	Tengs et al. (23)	95% CI, assumed 1 month
MI	0.1351	0.12	0.145	Bata	Sullivan et al. (22)	95% CI, ICD-9 410
GI bleeding	0.0486	0.030	0.060	Gamma	Earnshaw et al. (24)	95% CI
Disutility of DOAC	0.002	0.001	0.003	Gamma	Lip et al. (16)	95% CI
Disutility of warfarin	0.03	0.02	0.04	Gamma	Wang et al. (17), Zhao et al. (25)	95% CI
Disutility of aspirin	0.002	0.001	0.003	Gamma	Pink et al. (26), Canestaro et al. (27), Harrington et al. (28), Wang et al. (25), Zhao et al. (17)	95% CI
Disutility of dabigatran	0.03	0.02	0.04	Gamma	Wang et al. (25), Zhao et al. (17)	95% CI
Disutility of rivaroxaban	0.004	0.003	0.005	Gamma	Wang et al. (25), Zhao et al. (17)	95% CI
Disutility of apixaban	0.002	0.001	0.003	Gamma	Canestaro et al. (27), Wang et al. (25), Graham et al. (19), Zhao et al. (17)	95% CI
Disutility of edoxaban	0.002	0.001	0.003	Gamma	Canestaro et al. (27), Harrington et al. (28), Wang et al. (25), Zhao et al. (17)	95% CI
Disutility rate of utility in elderly cohort	0.997	–	–	–	Sullivan et al. (22)	–
**COST OF EVENTS**						
Major stroke	32,773.88	29,124.10	36,423.66	Gamma	Forrester et al. (29), Wang et al. (15)	95% CI
Minor stroke/ICH	20,732.65	18,424.53	23,041.85	Gamma	Forrester et al. (29), Wang et al. (15)	95% CI
Reversible stroke	12,459.01	11,072.61	13,847.58	Gamma	Forrester et al. (29), Wang et al. (15)	95% CI
Fatal stroke	11,947.50	9,558.00	14,337.00	Gamma	Reddy et al. (30)	Assumption range of ±20%, Distribution-assumed 20% *SD* of the mean
Major ICH	50,451.22	36,159.69	64,744.94	Gamma	Forrester et al. (29), Wang et al. (15)	95% CI
Fatal MI	9,367.902	7,494.322	11,241.48	Gamma	Reddy et al. (30)	Assumption range of ±20%, Distribution-assumed 20% *SD* of the mean
No fatal MI	6,312.528	5,050.022	7,575.034	Gamma	Reddy et al. (30)	Assumption range of ±20%, Distribution-assumed 20% *SD* of the mean
GIB	6,243.50	4,994.8	7,492.2	Gamma	Cholankeril et al. (31)	Assumption range of ±20%, Distribution-assumed 20% *SD* of the mean
**THE ANNUAL COST OF MAINTENANCE**						
Major stroke/ICH^*^	16,819.93	4,371.83	29,254.97	Gamma	Forrester et al. (29), Wang et al. (15)	95% CI
Minor stroke/ICH^*^	10,641.74	2,775.012	18,508.58	Gamma	Forrester et al. (29), Wang et al. (15)	95% CI
MI	1,014.37	928.69	1,097.28	Gamma	Tran et al. (32)	95% CI
Stroke and ICH	49,857.88	2,617.844	97,110.86	Gamma	Forrester et al. (29), Wang et al. (15)	95% CI
DOACs	3,342.31	2,154.24	4,956.51	LogNormal	Datar et al. (33)	95% CI, real-world pharmacy cost
Dabigatran	3,684.171	1,842.085	5,526.256	Gamma	Wang et al. (15)	National average drug acquisition costs, 95% CI
Rivaroxaban	3,852.662	1,926.331	5,778.994	Gamma	Wang et al. (15)	National average drug acquisition costs, 95% CI
Apixaban	3,856.893	1,928.447	5,785.34	Gamma	Wang et al. (15)	National average drug acquisition costs, 95% CI
Edoxaban	3,122.2	1,561.1	4,683.299	Gamma	Wang et al. (15)	National average drug acquisition costs, 95% CI
Aspirin	8.0886	1.99104	80.886	Gamma	Wang et al. (15)	National average drug acquisition costs, 95% CI
Warfarin and INR monitoring	492.24	78.52	798.41	LogNormal	Wang et al. (15)	95% CI

*AF, atrial fibrillation; GIB, gastrointestinal bleeding; ICH, intracranial hemorrhage; MI, myocardial infarction*.

* *Maintenance cost of stroke and ICH were assumed to be the same. All the costs were inflated to 2020 dollars*.

**Table 2:** Annual long-term death rate at given age.

**Death rate**	**Value (%/year)**	**lower(%/year)**	**upper(%/year)**	**Sources**
**Stroke**				
75	0.7386	0.722	0.7602	Global Burden of Disease Study 2017 (35)
80	1.2084	1.1805	1.2448	
85	1.8347	1.7975	1.891	
90	2.5217	2.4767	2.6037	
95	3.6216	3.5486	3.7492	
**Intracranial Hemorrhage**				
75	0.3457103	0.333784	0.333784	Global Burden of Disease Study 2017 (35)
80	0.4732861	0.4576446	0.4576446	
85	0.6637098	0.643405	0.643405	
90	0.8556525	0.8320234	0.8320234	
95	1.1019674	1.0716729	1.0716729	
**Myocardial Infarction**				
75	0.951	0.9339	0.9761	Global Burden of Disease Study 2017 (35)
80	1.6414	1.612	1.6858	
85	2.771	2.7278	2.8504	
90	4.4589	4.3902	4.5933	
95	7.2673	7.1337	7.503	

#### Health State Utilities and Cost

Health utility values, scored from 0 (death) to 1 (perfect health), were obtained from previous studies to describe the quality of life for each health state. The utility of 0.81 was reported for AF patients with an average age of 67 (22). Thus, the adjusted weight of 0.997 (age group >70 compared to 60–69) was further applied as a multiplying factor to calculate the baseline utilities of the cohort aged >75 years approximately (22). A permanent annual disutility of −0.2916, −0.4455, and −0.1351 were assumed for minor, major neurological events and MI. −0.0486 and −0.2916 were assessed as one-time dis-utilities for gastrointestinal bleeding (GIB) and the reversible stroke (23, 24). Anticoagulant therapies were also assumed to cause slight derogation of the health utilities, which were assessed to be from −0.002 to −0.03 for DOACs, warfarin, and Aspirin, respectively (Table 1) (16, 17, 25–28).

From the perspective of the US private payer, the model incorporated only direct healthcare costs for the therapies and treatments of the associated acute clinical events and the costs for long-term maintenance after experiencing the first non-fatal events. The costs of DOACs, warfarin, and aspirin were obtained from real-world pharmacy costs or the 2018 National Average Drug Acquisition cost in the US (Table 1). The clinical event costs were all obtained from the published literature and were inflated to reflect the 2020 value of the US dollar. Maintenance costs for major and minor neurological events were assumed to be similar, respectively. Annual maintenance costs for MI were derived from the average costs incurred during the 2–5-year period after diagnosis (32). All cost and utility inputs were discounted at 3 and 2% per annum to account for the effects of inflation and increasing economic valuation of health gains over time.

### Base-Case Analysis

The base-case analyses were conducted incorporating data of RCTs and OSs, respectively. The main outcomes of this analysis were the incremental costs, quality-adjusted life-years (QALYs) gained, and the incremental cost-effectiveness ratio (ICER). Cost-effectiveness was evaluated using the United States' conservative willingness-to-pay threshold of $50,000 and $100,000 per QALY (36). It was also assessed annually to determine the point at which the treatment options had achieved acceptable levels.

### Scenario and Sensitivity Analyses

Scenario, One-way sensitivity analysis, and the probabilistic sensitivity analysis (PSA) were performed to assess the impact of parameter uncertainty on the results. In one-way sensitivity analysis, the model parameters were varied over their 95% confidence intervals (CI). If the CI was not available, a variation of ±5% from the mean was assumed for the parameters of utility and ±20% for the parameters of probability and cost. In scenario analysis, the variations in the time horizons and the cost reduction of DOACs after patent expiry were conducted. In PSA, beta distributions were assumed for the clinical outcomes and health utilities, while gamma or log-normal distributions were assumed for the drug and healthcare costs. Hazard or risk ratios of DOACs compared to VKAs were assigned to beta or log-normal distributions. A 10,000-subject Monte Carlo simulation was conducted based on the variable distributions, and all the parameter inputs were allowed to vary stochastically in PSA. The PSA results are presented graphically as scatterplots.

## Results

### Base-Case Analysis

Over a 10-year projected time of a cohort with 10,000 patients, treatment with DOACs rather than with warfarin was predicted to result in fewer incidences of strokes, ICH, MI, and death, according to the simulation based on the real-world evidence (Table 3). Patients treated with warfarin were predicted to obtain 5.17 QALYs at the cost of $14,280.35, while treatment with DOACs resulted in 5.53 QALYs at the cost of $29,515.10. Therefore, the DOACs' additional benefit in reducing the number of total clinical events was associated with a gain of 0.36 QALYs at an additional cost of $15,234.65, resulting in an ICER of $42,318.47 per QALY. In the simulation incorporating the evidence of RCTs, a further reduction of ICH and death was predicted in both DOACs and warfarin but with increased stroke events. It is broadly consistent with the real practice because that regular follow-up of random control trials could help discover the asymptomatic stroke event but is more likely to screen out the patients with high bleeding risk by the rigorous inclusion and exclusion criteria. A slight rise of QALYs was predicted in RCTs than in OSs but with the increased ICER of $47,544.19 per QALY. It might due to the relatively higher drug cost of stroke compared to ICH, after which the patient is more likely to discontinue the therapy of anticoagulants and switch to aspirin (Table 3).

**Table 3:** Projected clinical events, costs, health benefits, and incremental ICER for base-case analysis over a 10-years life horizon in OSs and RCTs.

**Variables**	**DOACs (OSs)**	**Warfarin (OSs)**	**DOACs (RCTs)**	**Warfarin (RCTs)**
**No. of Events**				
Non-valvular atrial fibrillation	5,069	4,588	5,968	5,523
Minor stroke	165	176	318	364
Major stroke	168	186	327	383
Minor intracranial hemorrhage	153	300	54	106
Major intracranial hemorrhage	48	98	17	34
Stroke and intracranial hemorrhage	8	19	5	12
Myocardial infarction	128	136	60	63
Death	4,261	4,498	3,250	3,515
**Health Outcomes (Per Patient/10 Years)**				
QALYs (discounted)	5.53	5.17	5.94	5.57
Costs (discounted)	29,515.10	14,280.45	32,303.67	14,712.32
ICER ($/QALY)	42,318.47		47,544.19	

*DOACs, direct oral anticoagulants; QALY, quality-adjusted life-year; ICER, incremental cost-effectiveness ratio; NO, number; $, United States dollar*.

The total costs and QALYs of four individual DOACs (rivaroxaban, edoxaban, apixaban, and dabigatran) were also predicted. It indicated that the costs were lowest for warfarin ($14,280.45) and highest for rivaroxaban ($32,270.73). Edoxaban had the highest QALYs (6.04 QALYs), followed by apixaban, dabigatran, rivaroxaban, and warfarin. Compared to warfarin, the ICERs were $112,439.3, $71,587.32, $52,800.16, and $15,864.9 for rivaroxaban, dabigatran, apixaban, and edoxaban, indicating that edoxaban was the preferred therapy for stroke prevention in the elderly with AF.

### Scenario and One-Way Sensitivity Analysis

Variations in the time horizons were conducted in scenario analyses (Table 4). At 5-, 10-, and 15-year follow-ups, the ICER per QALY gained decreased from $55,451.94 to $33,301.50, indicating that long-term use of DOACs may provide additional benefits. The decrease of ICER was also predicted for dabigatran, edoxaban, and apixaban with the extension of the time horizon. However, a slight increase in cost-effectiveness estimates was obtained with the long-term use of rivaroxaban. This finding could be explained by the higher drug cost and moderate intensity in reducing the clinical events of rivaroxaban, leading to the lower health gain but increased overall costs.

**Table 4:** Base case analysis and one-way sensitivity analysis on the time horizon.

**Time horizon**	**Treatment therapies in the order of cost**			**Warfarin as the common reference**
	**Treatment strategies**	**Cost ($)**	**QALY**	**ICER ($/QALY)**
5-years	Warfarin	6,985.12	3.14	–
	Edoxaban	15,678.3	3.48	25,568.18
	DOACs	16,966.47	3.32	55,451.94
	Apixaban	18,042.55	3.3	69,108.94
	Dabigatran	18,481.24	3.23	127,734.67
	Rivaroxaban	18,927.41	3.25	108,566.27
10-years	Warfarin	14,280.45	5.17	–
	Edoxaban	28,082.91	6.04	15,864.9
	DOACs	29,515.1	5.53	42,318.47
	Apixaban	30,648.5	5.48	52,800.16
	Dabigatran	32,177.28	5.42	71,587.32
	Rivaroxaban	32,270.73	5.33	112,439.3
15-years	Warfarin	20,614.25	6.47	–
	Edoxaban	37,729.17	7.92	11,803.39
	DOACs	38,597.06	7.01	33,301.5
	Apixaban	39,332.22	6.9	43,530.16
	Rivaroxaban	41,501.56	6.66	109,933.2
	Dabigatran	42,145.25	6.9	50,072.09

*DOACs, direct oral anticoagulants; QALY, quality-adjusted life-year; ICER, incremental cost-effectiveness ratio; $, United States dollar*.

**Figure 2:**
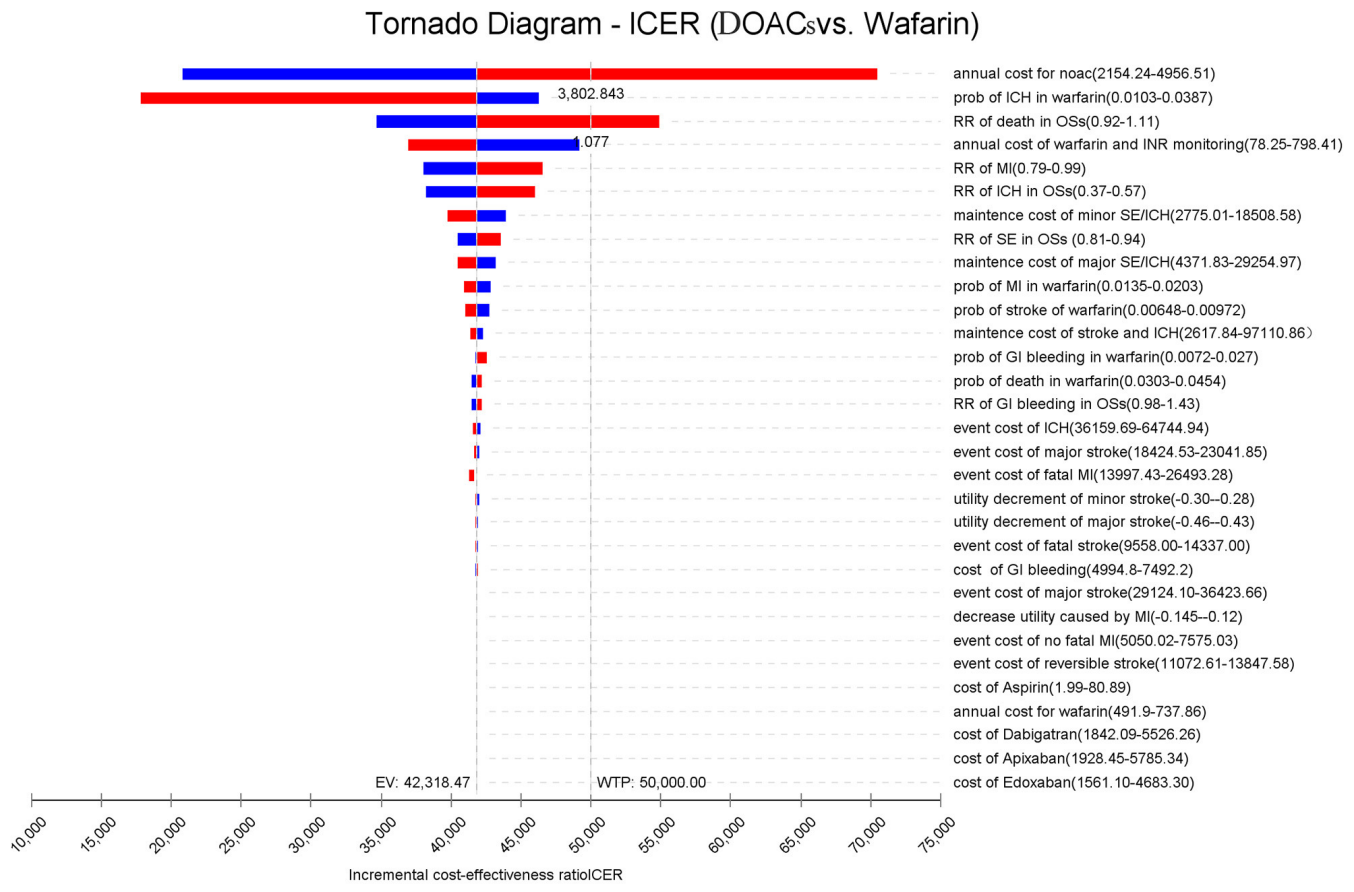
Scenario analysis on the cost of DOACs (Dabigatran, Rivaroxaban, Apixaban, and Edoxaban).

Figure 2 presents the sensitivity analyses of the vital parameter inputs that had the most significant impact on the ICERs, in the order of their respective influences. It was found that the cost of DOACs had an immense impact on the ICER, followed by the probability of ICH, the risk ratio of death, cost of warfarin and INR monitoring, and the risk ratio of MI. When the annual costs of DOACs were over $3,802.84 or the risk ratio of death was over 1.077%/year, the ICERs shifted from dominating to dominated status. As for the other vital parameters, the study found that varying the inputs across their plausible ranges resulted in changes in the ICER values. However, this had no significant effect on the results because DOACs was still more cost-effective than warfarin.

**Figure 3:**
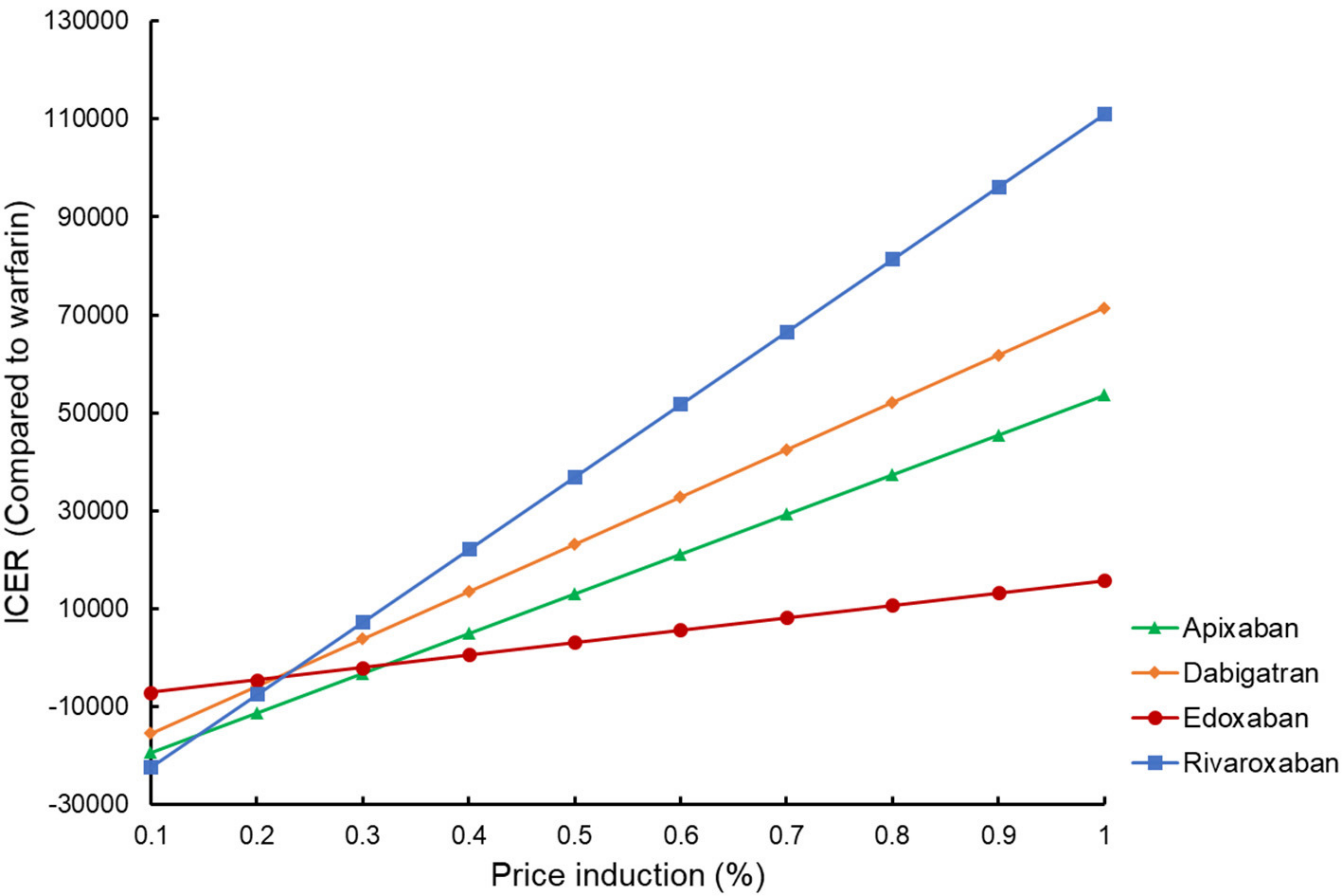
Tornado analysis: ICER of DOACs vs. warfarin over plausible ranges of model inputs.

By reducing the price of four DOACs from 10 to 90%, the potential impact of DOACs patent expiry on the outcomes was assessed (Figure 3). Rivaroxaban, dabigatran, and apixaban would become cost-effective compared to warfarin if their prices were cut down to 58.7, 77.7, and 95.5%, using a WTP threshold of $50,000/QALY. When $100,000/QALY was applied as a WTP threshold, apixaban and dabigatran would become cost-effective for stroke prevention in the elderly with AF, and about 92.5% of price reduction should be considered for rivaroxaban being undominated. Considering that the price of a given drug might drop by about 9–42% after the patent expiry in the competitive market (37), these four DOACs would probably become the preferred therapy for stroke prevention in the elderly population compared to warfarin in the further. In addition to the drug cost, deterministic sensitivity analysis showed that ICH event rate was another critical drive of the outcomes in the cost-effectiveness analysis between DOACs and warfarin. Using a WTP threshold of $50,000/QALY, dabigatran, rivaroxaban, and apixaban would be cost-effective compared to warfarin if the ICH rate dropped to <2.09, 3.33, and 1.47% per year, respectively. If there was a lower incidence of death and MI (<3.32 and 2.0% per year) or if the annual cost of warfarin exceeded $690.09, apixaban would shift to dominating status either.

### Probabilistic Sensitivity Analysis

**Figure 4:**
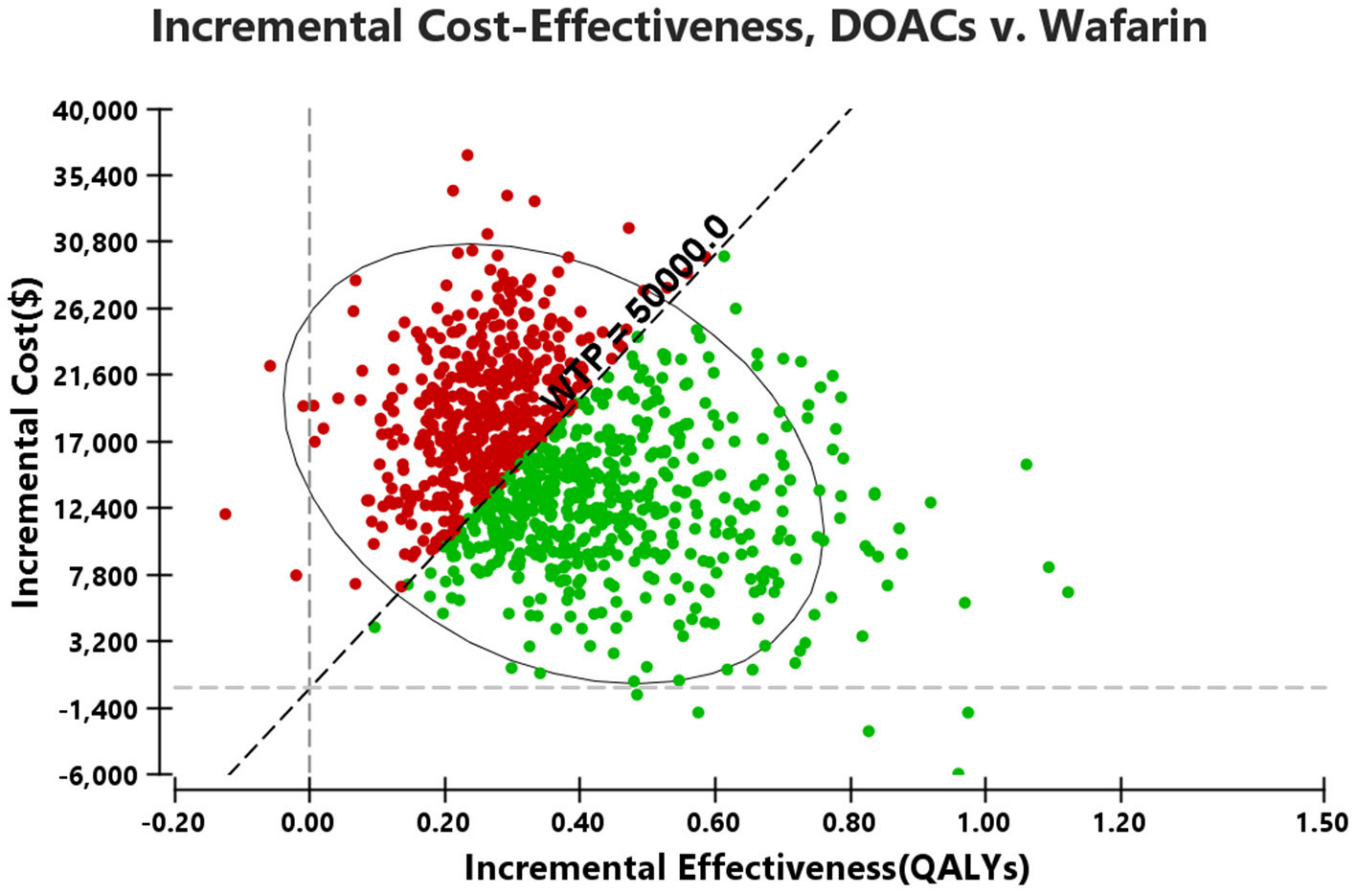
Results of the probabilistic sensitivity analyses.

Probabilistic sensitivity analysis simulations (Figure 4) demonstrated that over a 10-year life horizon, though requiring minimal additional costs, DOACs were more effective than warfarin. Treatment with DOACs resulted in a total cost of $29,493.82 (95% CI, 17,750.09–6,215.20), while the total cost of warfarin was estimated to be $14,021.90 (95% CI, 4,729.66–31,940.99). Over a 10-year horizon, DOACs gained an average QALY of 5.57 (95% CI, 4.18–6.79) vs. 5.21 (95% CI, 3.86–6.44) for warfarin (Table 5). The mean costs, QALYs, and ICERs of four DOACs were also estimated, and all the results were largely consistent with the base-case analysis (Table 5).

**Table 5:** Estimated costs, QALYs and ICER in PSA analyses on a 10-year time horizon.

**Treatment strategy**	**Therapies in the order of cost**		**Warfarin as the common reference**
	**Cost (95%CI)**	**QALY (95%CI)**	**ICER**
Warfarin	$14,021.90 (4,729.66–31,940.99)	5.21 (3.86–6.44)	0
Edoxaban	$27,352.70 (15,931.54–43,856.52)	6.09 (4.81–7.12)	$15,148.64
DOACs	$29,493.82 (17,750.09–6,215.20)	5.57 (4.18–6.79)	$42,977.56
Apixaban	$30,671.69 (16,849.47–48,239.88)	5.53 (4.01–6.81)	$52,030.59
Rivaroxaban	$32,179.39 (18,089.83–50,700.09)	5.41 (3.97–6.7)	$90,787.45
Dabigatran	$32,204.42 (18,392.88–50,856.49)	5.47 (4.14–6.62)	$69,932.76

*DOACs, direct oral anticoagulants; QALY, quality-adjusted life-year; ICER, incremental cost-effectiveness ratio; $, United States dollar*.

**Figure 5:**
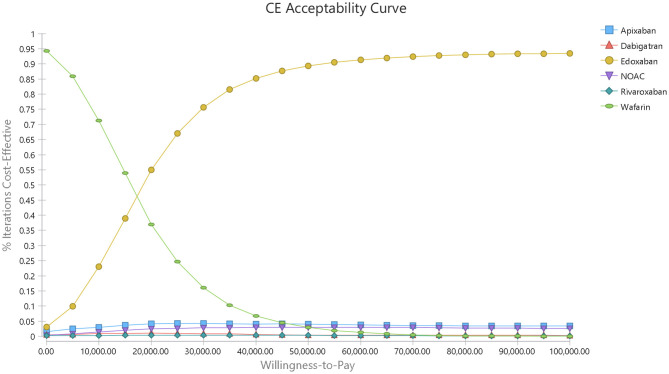
Willingness-to-pay curve.

A cost-effectiveness acceptability curve was plotted to demonstrate the proportion of simulations that were cost-effective at willingness-to-pay values (Figure 5). There was a 53.83 and 90.7% probability that DOACs were more cost-effective than warfarin when the willingness-to-pay threshold was set at $50,000 and $100,000/QALY, respectively. Compared to warfarin, dabigatran, apixaban, rivaroxaban, and edoxaban was cost-effective in 29.9, 44.27, 23.55, and 95.38% at the most conservative WTP of $50,000/QALY, and these probabilities arose to 59.37, 68.05, 52.91, and 99.40% correspondingly when the WTP of $100,000/QALY was applied (Supplementary Figure 1 and Figure 2).

## Discussion

### Main Findings and Interpretation

Based on a comprehensive overview of benefits and harms in real-life practice, this study provided a cost-effectiveness analysis and comparison of DOACs and warfarin in elderly AF patients from the perspective of the US private payer. The comparison of DOACs vs. warfarin in AF has been discussed frequently. Owing to their impressive efficacy and convincing safety profiles reported by RCTs, the economic viability of DOACs has been demonstrated in the US (9, 28), European countries (10, 11), and in some Asian countries (17, 38, 39). Despite their much higher costs, DOACs are generally more cost-effective than warfarin. However, the benefits of DOACs in the elderly population remain unclear as the elderly are generally perceived to have a high incidence of ICH, leading to a significant loss in quality of life and increased medical costs. In our previous work, a comprehensive systematic review and meta-analysis involving 27 high-quality observational studies (OSs) (519,267 patients) and three RCTs (28,152 patients) were conducted to estimate and compare the benefits and harms of DOACs and VKAs in the cohort aged >75 years (21). Compared with VKAs, DOACs significantly reduced the risk of stroke/SE and ICH without increasing the risk of GIB and all-cause mortality. In this study, based on the efficacy profiles derived from our previous work, we further conducted a comparative cost-effectiveness evaluation of DOACs (dabigatran, rivaroxaban, apixaban, and edoxaban) and VKAs in AF patients aged >75 years to thoroughly assess the expected costs and benefits of using DOACs in the elderly population. Major findings were as follows: (I) DOACs are more cost-effective treatment options than warfarin in real-world practice; (II) among all the four individual DOACs, edoxaban was the preferred treatment strategy by yielding the most significant health gain with the relatively low total cost.

### Comparison With Previous Studies

Until now, only one study has assessed the cost-effectiveness of DOACs in elderly AF patients (17). Although DOACs were generally more cost-effective than warfarin, it was concluded that the benefits appeared to be partially offset in elderly patients due to the increased bleeding risk. If the real-world risks of bleeding and ICH were factored in 4.8/1,000 person-years (40), the cost-effectiveness of DOACs relative to warfarin might end up being negated. However, only OSs on dabigatran and rivaroxaban were included in the above-mentioned study, resulting in underestimating the protective benefits of apixaban and edoxaban. In the present study, a cost-effectiveness evaluation was built upon a comprehensive meta-analysis of OSs that included all the available DOACs. Despite the high risk for major bleeding (about 13/1,000 person-years), which might be associated with the relatively loose inclusion and exclusion in OSs than in RCTs, it was concluded that DOACs was still more cost-effective than VKAs for elderly AF patients, In the sensitivity analysis, we further tripled the incidence of ICH and GIB. The ICER values did not increase over the cost-effectiveness threshold despite the significant increase in major bleeding risk, indicating that the treatment benefits of DOACs were of greater relevance and importance in older adults beyond the safety profile. In previous work, although all four individual DOACs were cost-effective compared to warfarin from the perspective of the private patient in Singapore, apixaban was found to be the preferred treatment strategy for AF patients aged over 75 years. While because of the relatively lower risk of all-cause death and MI, and the good protection effect in stroke prevention for elderly patients in real practice, edoxaban was revealed to be the optimal therapeutic strategy among all DOACs by yielding the highest health gain and comparatively lower total cost in the present study.

### Pivotal Factors for Cost-Effectiveness Analysis

In sensitivity analysis, drug cost was found to be one of the crucial factors in cost-effectiveness analysis. Since drug price reduction always happened for experiencing the generic entry after the patent protection, the potential affordability of DOACs compared to warfarin after patent expiry was further assessed in scenario analysis. Using a most conservative WTP threshold of $50,000/QALY, rivaroxaban, apixaban, and edoxaban, the three dominated DOACs with the current prices, could become cost-effective only by a slight to a moderate price reduction <50%). As the price of a given drug might drop by about 9–42% after the patent expiry (37), it is plausible to suppose that all the four DOACs would become the preferred therapy in comparison with warfarin for the stroke prevention in the elderly population in the further.

## Strengths and Limitations

The main strength of this study was that using data from OSs, it compared the cost-effectiveness of all available DOACs (dabigatran, rivaroxaban, apixaban, and edoxaban) and warfarin in elderly AF patients. However, several intrinsic limitations should be addressed in this study. First, the rates of clinical events in this model were derived from OSs that had follow-up durations of up to 2.8 years. It was likely to produce a bias by extrapolating the event rates to a 10-year time horizon. Second, although medical costs are generally high for elderly patients, cost increases associated with age were not included in this study due to the lack of accurate cost information. Third, the results of this work were subject to several assumptions, such as the switch to aspirin after any ICH event, the transition to death after two major neurological events, and so on. These assumptions may help to approximate the AF disease progression in real-world practice but would probably produce conservative results to a certain extent. Fourth, specific treatment effects associated with the dosage of DOACs (including off-label and the low dosage), time-in-therapeutic ranges of warfarin, poor compliance in elderly patients, and the switch among different DOACs are not noted due to the lack of specific data in available OSs. In fact, because of the poor kidney function or some other risk of bleeding in the elderly, the reduced-dose DOACs were widely used in most populations. And the poor compliance in frail elderly patients complicates the management of antithrombotic therapy further. Time-in-therapeutic ranges of warfarin should also be noted, as they are closely related to the treatment effect of the warfarin. Thus, the results of our analysis may be conservative because of the deviations of drug administration in real practice.

## Conclusions

For the elderly with AF, despite the high risk for major bleeding, DOACs are more cost-effective treatment options than warfarin in real-world practice. Edoxaban was the preferred treatment strategy among four kinds of DOACs for AF patients aged over 75 years. The treatment benefits of DOACs assumed greater relevance and importance in older adults beyond the safety profile.

## Data Availability Statement

The original contributions presented in the study are included in the article/Supplementary Material, further inquiries can be directed to the corresponding author/s.

## Author Contributions

Z-CG is the guarantor of the entire manuscript. Z-CG and YW contributed to the study conception and design and critical revision of the manuscript for important intellectual content. CZ contributed to data acquisition, analysis, and interpretation. All authors contributed to the article and approved the submitted version.

## Conflict of Interest

The authors declare that the research was conducted in the absence of any commercial or financial relationships that could be construed as a potential conflict of interest.
