# Supplementary material for: Cost-Effectiveness Analysis of Direct Oral Anticoagulants Vs. Vitamin K Antagonists in the Elderly With Atrial Fibrillation: Insights From the Evidence in a Real-World Setting
Source: Front Cardiovasc Med. 2021 Jun 29;8:675200. doi: 10.3389/fcvm.2021.675200 (PMC8275875; doi:10.3389/fcvm.2021.675200)
Supplement: Supplementary file 1 [file Data_Sheet_1.pdf]

Supplementary Table 1. Relative risk of Stroke, ICH, GIB, mortality and MI in dabigatran, rivaroxaban, apixaban, edoxaban and aspirin compared to warfarin

Parameters	Base-case value	Low	Up	Distribution	Sources	Notes
**Probability**						
Stroke (warfarin) in RCTs	0.015	0.01	0.02	Beta	(Connolly et al.; Granger et al.; Patel et al.; Giugliano et al.)(Connolly et al., 2009; Granger et al., 2011; Patel et al., 2011; Giugliano et al., 2013)	Meta-analysis of RCTs; 95% CI
ICH (warfarin) in RCTs	0.004	0.003	0.006	Beta	(Connolly et al.; Granger et al.; Patel et al.; Giugliano et al.)(Connolly et al., 2009; Granger et al., 2011; Patel et al., 2011; Giugliano et al., 2013)	Meta-analysis of RCTs; 95% CI
MI (warfarin) in RCTs	0.007	0.005	0.009	Beta	(Connolly et al.; Granger et al.; Patel et al.; Giugliano et al.)(Connolly et al., 2009; Granger et al., 2011; Patel et al., 2011; Giugliano et al., 2013)	Meta-analysis of RCTs; 95% CI
GI bleeding (warfarin) in RCTs	0.011	0.008	0.015	Beta	(Connolly et al.; Granger et al.; Giugliano et al.)(Connolly et al., 2009; Granger et al., 2011; Giugliano et al., 2013)	Meta-analysis of RCTs; 95% CI

Mortality (warfarin) in RCTs	0.033	0.025	0.042	Beta	(Connolly et al.; Granger et al.; Patel et al.; Giugliano et al.)(Connolly et al., 2009; Granger et al., 2011; Patel et al., 2011; Giugliano et al., 2013)	Meta-analysis of RCTs; 95% CI
**Relative risk of Stroke in NOACs relative to warfarin in OSs**						
Dabigatran	0.9	0.83	0.98	-	Shen et al.(Shen et al., 2020)	Meta-analysis of real word; 95% CI
Rivaroxaban	0.85	0.76	0.94	-		
Apixaban	0.74	0.51	0.98	-		
Edoxaban	0.69	0.48	0.89	-		
Aspirin	2.08	1.59	2.70	-	van Walraven et al.(van Walraven et al., 2002)	95% CI
**Relative risk of ICH in NOACs relative to warfarin in OSs**						
Dabigatran	0.42	0.28	0.56	-	Shen et al.(Shen et al., 2020)	Meta-analysis of real word; 95% CI
Rivaroxaban	0.60	0.32	0.87	-		

Apixaban	0.35	0.17	0.69	-		
Edoxaban	0.30	0.06	0.53	-		
Aspirin	0.51	0.16	1.60		Roskell et al.(Roskell et al., 2010)	95% CI
**Relative risk of GIB in NOACs relative to warfarin in OSs**						
Dabigatran	1.48	1.23	1.72	-	Shen et al.(Shen et al., 2020)	Meta-analysis of real word; 95% CI
Rivaroxaban	1.08	0.71	1.45	-		
Apixaban	0.21	0.09	0.46	-		
Edoxaban	0.53	0.1	0.95	-		
Aspirin	0.63	0.32	1.22	-	Roskell et al.(Roskell et al., 2010)	95% CI
**Relative risk of mortality in NOACs relative to warfarin in OSs**						
Dabigatran	0.94	0.82	1.05	-	Shen et al.(Shen et al., 2020)	Meta-analysis of real word; 95% CI
Rivaroxaban	1.15	0.93	1.37	-		

Apixaban	1.21	0.95	1.47	-		
Edoxaban	0.73	0.52	0.98	-		
**Relative risk of MI in NOACs relative to warfarin in OSs**						
Dabigatran	0.87	0.73	1.01	-	Shen et al.(Shen et al., 2020)	Meta-analysis of real word; 95% CI
Rivaroxaban	0.96	0.79	1.13	-		
Apixaban	0.67	0.32	1.41	-		
Edoxaban	0.52	0.2	1.32	-		
Aspirin	1.42	0.84	2.39		Roskell et al.(Roskell et al., 2010)	95% CI

GIB, gastrointestinal bleeding; ICH, intracranial hemorrhage; MI, myocardial infarction.

Supplementary Table 2. The raw data of Figure 5

WEIGHT	STRATEGY	STRATEGYNAME	ACCEPTABILITY
0	4	Apixaban	0.0153
5000	4	Apixaban	0.0245
10000	4	Apixaban	0.0302
15000	4	Apixaban	0.0369
20000	4	Apixaban	0.0416
25000	4	Apixaban	0.0429
30000	4	Apixaban	0.0423
35000	4	Apixaban	0.041
40000	4	Apixaban	0.0408
45000	4	Apixaban	0.0409
50000	4	Apixaban	0.0402
55000	4	Apixaban	0.0393
60000	4	Apixaban	0.0379
65000	4	Apixaban	0.0362
70000	4	Apixaban	0.0359
75000	4	Apixaban	0.0351
80000	4	Apixaban	0.0349
85000	4	Apixaban	0.0339
90000	4	Apixaban	0.0342
95000	4	Apixaban	0.0343
100000	4	Apixaban	0.034
0	2	Dabigatran	0.0046
5000	2	Dabigatran	0.0064
10000	2	Dabigatran	0.0091
15000	2	Dabigatran	0.01
20000	2	Dabigatran	0.011

25000	2	Dabigatran	0.0094
30000	2	Dabigatran	0.0089
35000	2	Dabigatran	0.0083
40000	2	Dabigatran	0.0059
45000	2	Dabigatran	0.0045
50000	2	Dabigatran	0.004
55000	2	Dabigatran	0.0036
60000	2	Dabigatran	0.0039
65000	2	Dabigatran	0.0041
70000	2	Dabigatran	0.0041
75000	2	Dabigatran	0.0039
80000	2	Dabigatran	0.0037
85000	2	Dabigatran	0.0035
90000	2	Dabigatran	0.0034
95000	2	Dabigatran	0.0032
100000	2	Dabigatran	0.0031
0	5	Edoxaban	0.031
5000	5	Edoxaban	0.0991
10000	5	Edoxaban	0.2304
15000	5	Edoxaban	0.3899
20000	5	Edoxaban	0.5495
25000	5	Edoxaban	0.6699
30000	5	Edoxaban	0.7565
35000	5	Edoxaban	0.8149
40000	5	Edoxaban	0.8522
45000	5	Edoxaban	0.8763
50000	5	Edoxaban	0.8931
55000	5	Edoxaban	0.9055
60000	5	Edoxaban	0.9128

65000	5	Edoxaban	0.9189
70000	5	Edoxaban	0.9239
75000	5	Edoxaban	0.9276
80000	5	Edoxaban	0.93
85000	5	Edoxaban	0.9324
90000	5	Edoxaban	0.9328
95000	5	Edoxaban	0.9337
100000	5	Edoxaban	0.9347
0	0	DOAC	0.0038
5000	0	DOAC	0.0085
10000	0	DOAC	0.0145
15000	0	DOAC	0.0198
20000	0	DOAC	0.0251
25000	0	DOAC	0.0266
30000	0	DOAC	0.0284
35000	0	DOAC	0.029
40000	0	DOAC	0.0298
45000	0	DOAC	0.0296
50000	0	DOAC	0.0301
55000	0	DOAC	0.0302
60000	0	DOAC	0.0302
65000	0	DOAC	0.03
70000	0	DOAC	0.0292
75000	0	DOAC	0.0285
80000	0	DOAC	0.0279
85000	0	DOAC	0.027
90000	0	DOAC	0.027
95000	0	DOAC	0.0265
100000	0	DOAC	0.0261

0	3	Rivaroxaban	0.002
5000	3	Rivaroxaban	0.0022
10000	3	Rivaroxaban	0.0031
15000	3	Rivaroxaban	0.0039
20000	3	Rivaroxaban	0.0037
25000	3	Rivaroxaban	0.0042
30000	3	Rivaroxaban	0.0038
35000	3	Rivaroxaban	0.0039
40000	3	Rivaroxaban	0.0039
45000	3	Rivaroxaban	0.0039
50000	3	Rivaroxaban	0.0033
55000	3	Rivaroxaban	0.0028
60000	3	Rivaroxaban	0.0026
65000	3	Rivaroxaban	0.0026
70000	3	Rivaroxaban	0.002
75000	3	Rivaroxaban	0.0018
80000	3	Rivaroxaban	0.0018
85000	3	Rivaroxaban	0.0019
90000	3	Rivaroxaban	0.0018
95000	3	Rivaroxaban	0.0016
100000	3	Rivaroxaban	0.0016
0	1	Warfarin	0.9433
5000	1	Warfarin	0.8593
10000	1	Warfarin	0.7127
15000	1	Warfarin	0.5395
20000	1	Warfarin	0.3691
25000	1	Warfarin	0.247
30000	1	Warfarin	0.1601
35000	1	Warfarin	0.1029

40000	1	Warfarin	0.0674
45000	1	Warfarin	0.0448
50000	1	Warfarin	0.0293
55000	1	Warfarin	0.0186
60000	1	Warfarin	0.0126
65000	1	Warfarin	0.0082
70000	1	Warfarin	0.0049
75000	1	Warfarin	0.0031
80000	1	Warfarin	0.0017
85000	1	Warfarin	0.0013
90000	1	Warfarin	0.0008
95000	1	Warfarin	0.0007
100000	1	Warfarin	0.0005

Supplementary figure 1. Incremental CE Scatter Plot of individual DOACs vs warfarin at WTP of $50,000/QALY

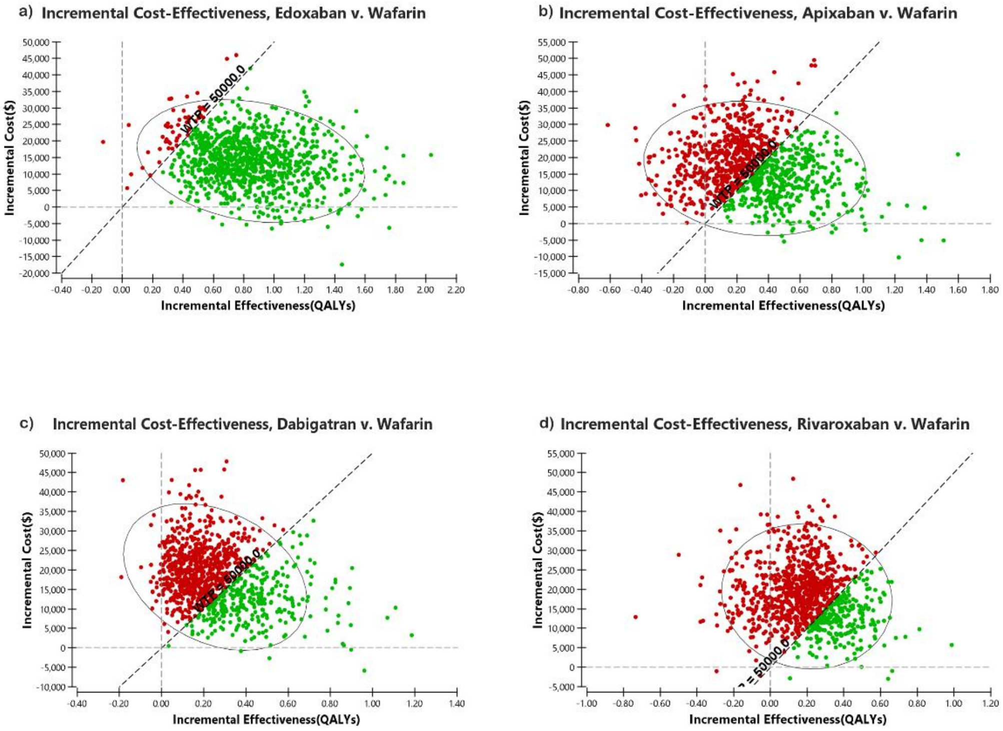

Supplementary figure 2. Incremental CE Scatter Plot of individual DOACs vs warfarin at WTP of $100,000/QALY

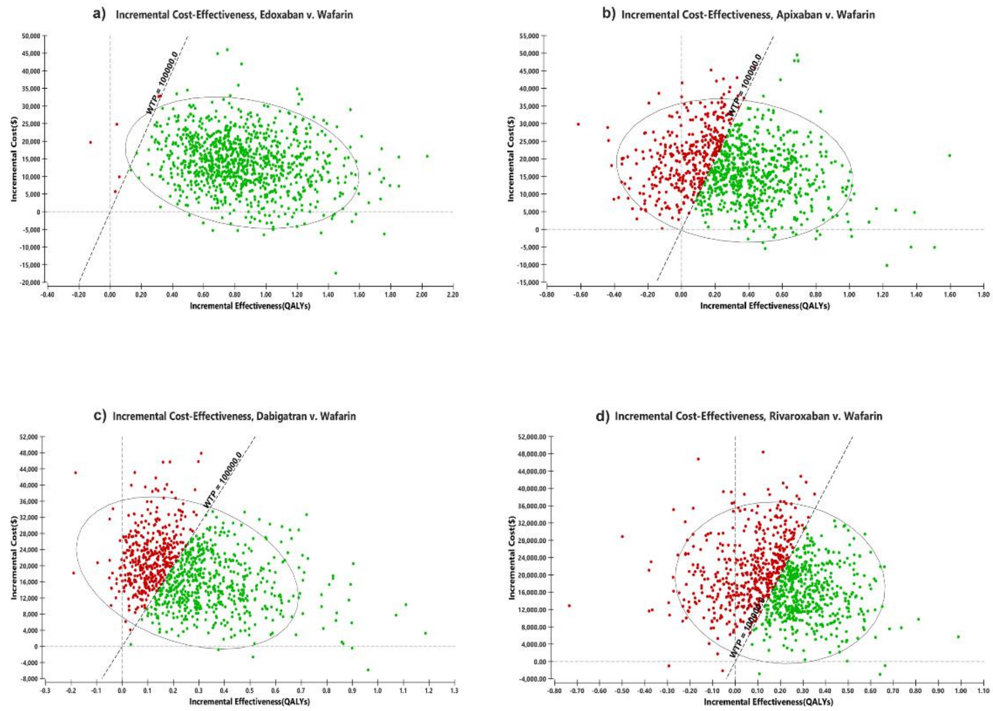

References

Connolly, S.J., Ezekowitz, M.D., Yusuf, S., Eikelboom, J., Oldgren, J., Parekh, A., et al. (2009). Dabigatran versus warfarin in patients with atrial fibrillation. *N Engl J Med* 361(12)**,** 1139-1151. doi: 10.1056/NEJMoa0905561.

Giugliano, R.P., Ruff, C.T., Braunwald, E., Murphy, S.A., Wiviott, S.D., Halperin, J.L., et al. (2013). Edoxaban versus warfarin in patients with atrial fibrillation. *N Engl J Med* 369(22)**,** 2093-2104. doi: 10.1056/NEJMoa1310907.

Granger, C.B., Alexander, J.H., McMurray, J.J., Lopes, R.D., Hylek, E.M., Hanna, M., et al. (2011). Apixaban versus warfarin in patients with atrial fibrillation. *N Engl J Med* 365(11)**,** 981-992. doi: 10.1056/NEJMoa1107039.

Patel, M.R., Mahaffey, K.W., Garg, J., Pan, G., Singer, D.E., Hacke, W., et al. (2011). Rivaroxaban versus warfarin in nonvalvular atrial fibrillation. *N Engl J Med* 365(10)**,** 883-891. doi:

10.1056/NEJMoa1009638.

Roskell, N.S., Lip, G.Y., Noack, H., Clemens, A., and Plumb, J.M. (2010). Treatments for stroke prevention in atrial fibrillation: a network meta-analysis and indirect comparisons versus dabigatran etexilate. *Thromb Haemost* 104(6)**,** 1106-1115. doi: 10.1160/th10-10-0642.

Shen, N.-N., Wu, Y., Wang, N., Kong, L.-C., Zhang, C., Wang, J.-L., et al. (2020). Direct Oral Anticoagulants vs. Vitamin-K Antagonists in the Elderly With Atrial Fibrillation: A Systematic Review Comparing Benefits and Harms Between Observational Studies and Randomized Controlled Trials. *Frontiers in cardiovascular medicine* 7**,** 132-132. doi: 10.3389/fcvm.2020.00132.

van Walraven, C., Hart, R.G., Singer, D.E., Laupacis, A., Connolly, S., Petersen, P., et al. (2002). Oral anticoagulants vs aspirin in nonvalvular atrial fibrillation: an individual patient meta-analysis. *Jama* 288(19)**,** 2441-2448. doi: 10.1001/jama.288.19.2441.
